# Microstructure and Dielectric Properties of LPCVD/CVI-SiBCN Ceramics Annealed at Different Temperatures

**DOI:** 10.3390/ma10060655

**Published:** 2017-06-15

**Authors:** Jianping Li, Mingxi Zhao, Yongsheng Liu, Nan Chai, Fang Ye, Hailong Qin, Laifei Cheng, Litong Zhang

**Affiliations:** 1 Science and Technology on Thermostructural Composite Materials Laboratory, Northwestern Polytechnical University, Xi’an 710072, China; ijianping@163.com (J.L.); mingxizhao@mail.nwpu.edu.cn (M.Z.); chainan.90@163.com (N.C.); yefang511@nwpu.edu.cn (F.Y.); qinxlong555@163.com (H.Q.); chenglf@nwpu.edu.cn (L.C.); zhanglt@nwpu.edu.cn (L.Z.); 2 State Key Laboratory of Solidification Processing, Northwestern Polytechnical University, Xi’an 710072, China

**Keywords:** siliconboron carbonitride ceramic, dielectric properties, electromagnetic wave absorbing properties, heat treatment, chemical vapor deposition and infiltration

## Abstract

SiBCN ceramics were introduced into porous Si_3_N_4_ ceramics via a low-pressure chemical vapor deposition and infiltration (LPCVD/CVI) technique, and then the composite ceramics were heat-treated from 1400 °C to 1700 °C in a N_2_ atmosphere. The effects of annealing temperatures on microstructure, phase evolution, dielectric properties of SiBCN ceramics were investigated. The results revealed that α-Si_3_N_4_ and free carbon were separated below 1700 °C, and then SiC grains formed in the SiBCN ceramic matrix after annealing at 1700 °C through a phase-reaction between free carbon and α-Si_3_N_4_. The average dielectric loss of composites increased from 0 to 0.03 due to the formation of dispersive SiC grains and the increase of grain boundaries.

## 1. Introduction

Siliconboron carbonitride ceramics (SiBCN) are considered as promising materials for high-temperature structural ceramics, continuous fiber reinforced ceramic matrix composites (CFCC) [1,2,3,4,5], and agents for electromagnetic wave (EMW) absorber [6], etc., because of their low density, supreme ultra-high temperature stability up to 2000 °C [7], good resistance to oxidation and creep [8], and high strength and modulus [7,8,9]. More importantly, with the development of stealth, radar-absorbing technology, and telecommunications, the study of EMW absorption has gained worldwide interest [10,11,12]. For non-magnetic ceramic materials, ideal EMW absorption materials must satisfy two requirements: (1) the impedance matching between free space and the material surface to prevent the wave being reflected, which requires the complex permittivity to be close to 1; and (2) materials can absorb as many incident waves as possible inside of absorbers, which requires materials exhibit strong dielectric loss [13]. In the quaternary SiBCN ceramics, SiC is a wide band gap semiconductor material and carbon is a good conductor, both of which have excellent EMW absorption and shielding properties and can be good dielectric loss phases [12,14,15]. Si_3_N_4_ and BN have low dielectric constants and dielectric losses, which can be regarded as wave-transparent matrices and impedance matching phases. Therefore, SiBCN ceramics have aroused much enthusiasm for high-temperature functional materials not only because of the excellent EMW absorption property but also due to the controllable EMW absorption property by designing phase composition and microstructure [16,17].

Recently, many methods have been developed to prepare different types of SiBCN ceramics, including the polymer pyrolyzing route [18], chemical vapor deposition and infiltration (CVD/CVI) [19,20], reactive magnetron sputtering [21], and a mechanical alloying plus sintering technique [22]. EMW-absorbing properties of SiBCN ceramics made by polymer pyrolyzing and CVD/CVI was studied. Ye et al. [16] fabricated SiBCN ceramics with extremely low dielectric loss (about 0.01) via polymer-derived ceramics (PDCs), while their dielectric property is improved significantly when annealed above 1650 °C owing to the crystallization of SiC grains and structural transformation. The element content and phase composition of ceramics fabricated via PDCs can be adjusted by controlling the structure of the precursor. Wang et al. [23] found that β-Si_3_N_4_ and β-SiC were separated when a novel SiBCN ceramic was annealed at 2200 °C in N_2_ atmosphere. Therefore, the structural transformation of SiBCN ceramics after annealing at high temperature may have a positive impact on their dielectric and EMW absorbing capabilities.

In our previous work, low-pressure chemical vapor deposition/infiltration (LPCVD/CVI) SiBCN ceramics, which have been designed by thermodynamic calculation, have various and complex phases, such as Si_3_N_4_+SiC+C+BN, SiC+C+B and B_4_C+SiC+C+BN, when using a gas mixture of CH_3_SiCl_3_-NH_3_-BCl_3_-H_2_ and the phase content can be controlled by adjusting experimental conditions [20]. In our previous researches, LPCVD/CVI technology was used for the first time to introduce SiBCN ceramics into porous Si_3_N_4_ substrates [24]. The obtained SiBCN ceramic was amorphous and SiBCN-Si_3_N_4_ composite ceramics had a dielectric loss of 0 when fabricated at 800 °C and 0.1 at 900 °C, exhibiting tunable EMW absorbing capability [24]. 

Generally, high temperature has an important effect on microstructure, phase composition, and properties of ceramic materials. On the one hand, heat treatment is frequently used as the process to improve the functional properties of ceramic materials [16,25]; on the other hand, the microstructure and properties of ceramics might change when used in high-temperature fields. Therefore, it is necessary to investigate the microstructure evolution and impact of separated phases on the dielectric and EMW absorption properties of LPCVD/CVI SiBCN ceramics. Focusing on this purpose, in the present work, the LPCVD/CVI SiBCN ceramics were prepared on a porous Si_3_N_4_ matrix at 800 °C with the same deposition parameters as those in our previous study [24], and then these composite ceramics were annealed at high temperatures in a nitrogen atmosphere. The thermodynamic diagram demonstrated the SiBCN consisted of Si_3_N_4_+SiC+C+BN [24]. The effect of the annealing temperature on the microstructure, phase evolution, thermal stability, dielectric, and EMW absorption properties were investigated.

## 2. Materials and Methods

Si_3_N_4_ ceramics with porosity of about 30% were used as deposition substrates. The Si_3_N_4_ ceramics fabricated using the previous method [26] were machined into specimens with dimensions of 2.16 mm × 10.16 mm × 22.86 mm for dielectric properties measurement. Then these samples were put into a CVD/CVI furnace to infiltrate SiBCN ceramics using methyltrichlorosilane (CH_3_SiCl_3_, MTS ≥ 99.99%), boron trichloride (BCl_3_ ≥ 99.99%), and ammonia (NH_3_ ≥ 99.99%) as gas resources at 800 °C. The as-received composites were termed as AS. Hydrogen (H_2_ ≥ 99.99%) was used as the carrier gas of MTS and the dilution gas. Argon (Ar ≥ 99.9%) was also used as the dilution gas. Finally, these samples were heat-treated at 1400, 1500, 1600, and 1700 °C in a high-purity N_2_ atmosphere for 2 h (N_2_, 0.3 MPa) using a hot-pressing furnace (High-Multi 5000, Fujidempa Kogyo, Osaka, Japan), and the obtained composite ceramics were named HT-14, HT-15, HT-16, and HT-17, respectively.

Weight loss of SiBCN-Si_3_N_4_ composite ceramics after annealing was measured by an electronic balance (Mettler Toledo, AG204) with precision of 0.0001 g. The microstructure and phase composition were analyzed by scanning electron microscopy (SEM; S-4700; Hitachi, Tokyo, Japan) and X-ray diffraction (XRD; D8-Advance, Bruker, Karlsruhe, Germany), respectively. X-ray photoelectron spectrometry (XPS, K-Alpha; Thermo Scientific, Waltham, MA, USA) was carried out to verify the bonding structure. Raman spectra were recorded on a Renishaw Ramoscope (confocal Raman microscope, inVia, Renishaw, Gloucestershire, UK) equipped with a He-Ne laser (λ = 514.5 nm). The complex permittivity for X-band was measured via a vector network analyzer (VNA, MS4644A; Anritsu, Atsugi, Japan) using the waveguide method.

## 3. Results and Discussion

**Figure 1:**
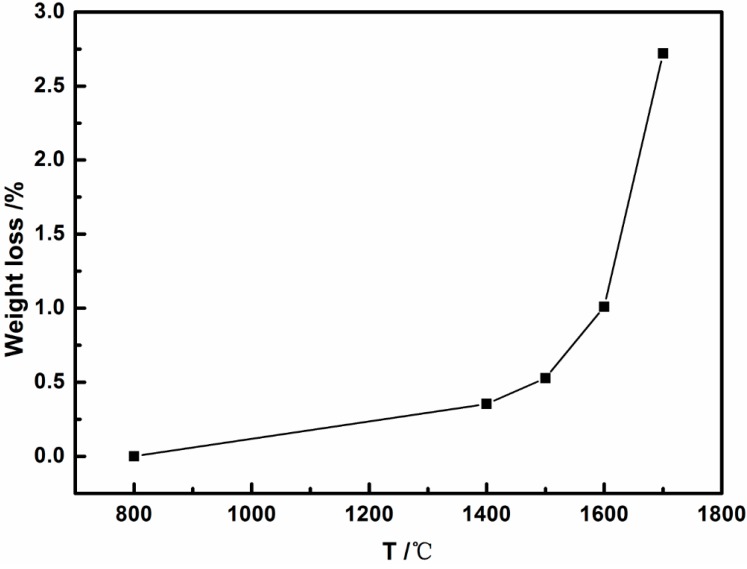
Weight loss of SiBCN-Si_3_N_4_ composite ceramics after annealing at different temperatures.

**Figure 2:**
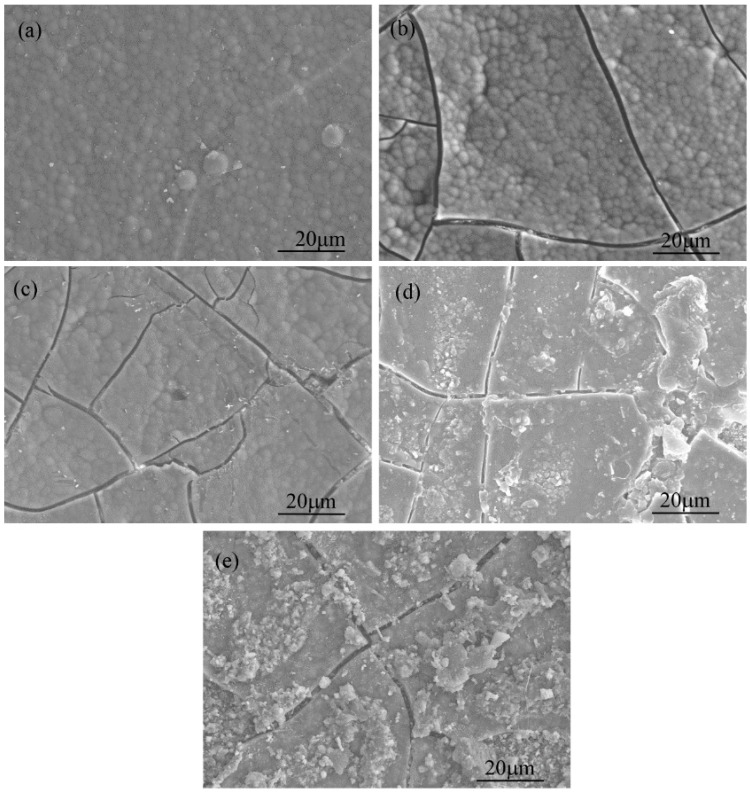
The surface morphology of as-received (**a**) and annealed SiBCN-Si_3_N_4_ ceramics at different temperatures: (**b**) HT-14; (**c**) HT-15; (**d**) HT-16; and (**e**) HT-17.

As shown in Figure 1, the weight loss of SiBCN-Si_3_N_4_ composite ceramics increased slightly as the annealing temperature increased. However, a remarkable weight loss of 2.72% was found when the annealing temperature reached 1700 °C, which indicated that there were some reactions occurred, and this may lead to structure transformation or decomposition of SiBCN ceramics. It will be confirmed by our following analysis. The surface morphologies of as-received and annealed SiBCN-Si_3_N_4_ composite ceramics at different temperatures ranging from 1400 °C to 1700 °C are presented in Figure 2. As we can see from Figure 2a, the as-received SiBCN ceramics via LPCVD/CVI were amorphous with a compact and continuous cauliflower-like surface morphology. However, due to the mismatch in thermal expansion coefficients between SiBCN and Si_3_N_4_, a large amount of cracks appeared uniformly on the SiBCN coating for all annealed samples. When the temperature was up to 1600 °C, some particles formed in ceramics, which might be attributed to the crystallization of ceramics, and this will be proved by several methods in the following part of the paper. With the further increase of temperature to 1700 °C, more particles and flocculent products covered the surface of SiBCN-Si_3_N_4_ ceramics. The various surface morphologies at different temperatures indicated that the process of crystallization was strongly influenced by the increase of annealing temperature.

**Figure 3:**
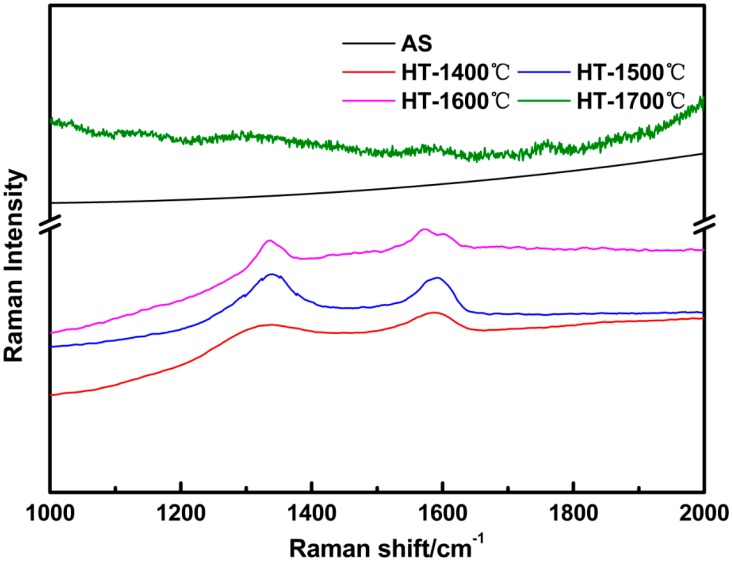
Raman spectra of as-received and annealed SiBCN-Si_3_N_4_ ceramics at different temperatures.

The Raman spectroscopy technique, with high sensitivity to carbon, was performed to record the structure evolution of free carbon within SiBCN ceramics before and after annealing from 1400 °C to 1700 °C. As shown in Figure 3, there were no bands observed from the spectra of as-received SiBCN ceramic in the whole frequency region, suggesting extremely low free carbon content. After annealing at 1400 °C, two broad signals centered around 1350 cm^−1^ and 1582 cm^−1^ appeared and maintained existence to 1600 °C, which were representative features of the free carbon termed D band and G band, respectively. The D and G bands can vary in intensity, position, and width depending on the structural organization of the samples. The intensity ratio of the D and G modes (I(D)/I(G)) can predict the ordering and carbon cluster size of free carbon. The I(D)/I(G) of HT-14, HT-15, and HT-16 were 1.27, 1.27, and 1.19, respectively, implying the free carbon underwent the conversion from amorphous to nanocrystalline graphite when the temperature increased to 1600 °C, according to the three-stage model reported by Ferrari and Robertson [11,27,28]. When the temperature further increased to 1700 °C, there were no D and G bands, which may indicate that the free carbon was consumed to an extremely low content by chemical reactions occurring in the SiBCN ceramic when annealed at 1700 °C. This was confirmed by the following XRD and XPS analysis.

**Figure 4:**
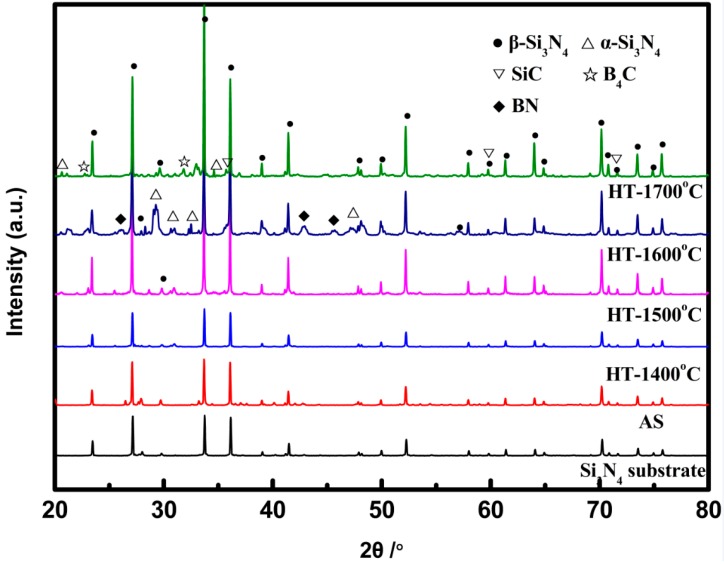
X-ray diffraction patterns of SiBCN-Si_3_N_4_ ceramics with different heat-treatment temperatures.

The crystallization behavior of SiBCN-Si_3_N_4_ composite ceramics was studied through X-ray diffraction (Figure 4). The spectra of as-received SiBCN ceramic was the same with that of Si_3_N_4_ substrate which consists of β-Si_3_N_4_, indicating the amorphous state of as-received SiBCN ceramics. When the temperature increased from 1500 °C to 1700 °C, there were β-SiC peaks in annealed SiBCN and the intensity increased with the increasing temperature. However, the case for α-Si_3_N_4_ and BN is quite different. The characteristic peaks of α-Si_3_N_4_ appeared after annealing at 1600 °C, which suggested the crystallization of α-Si_3_N_4_ occurred. Whereas some of characteristic peaks of α-Si_3_N_4_ disappeared and the intensity of some decreased after annealing at 1700 °C, indicating the dropped amount of α-Si_3_N_4_. Additionally, BN showed the same trend that it crystallized at 1600 °C and disappeared at 1700 °C. Therefore, on the basis of XRD and Raman results, it can be concluded that the crystallization phases were Si_3_N_4_+SiC+C+BN and were consistent with the thermodynamic diagram [24]. Nevertheless, B_4_C was found in SiBCN ceramic when annealing at 1700 °C, coupled with decreased intensity of α-Si_3_N_4_ and disappearances of carbon peaks in Raman spectra, which might indicate that chemical reactions occurred in SiBCN ceramic when annealed at 1700 °C. 

In terms of the above results, it can be inferred that chemical reaction occurred in SiBCN ceramic when annealed at 1700 °C, and it was expressed as the following equations:

(1) $$4\mathrm{BN}+C\to B_{4}C+2N_{2}$$

(2) $${\mathrm{Si}}_{3}N_{4}+3C\to 3\mathrm{SiC}+2N_{2}$$

**Figure 5:**
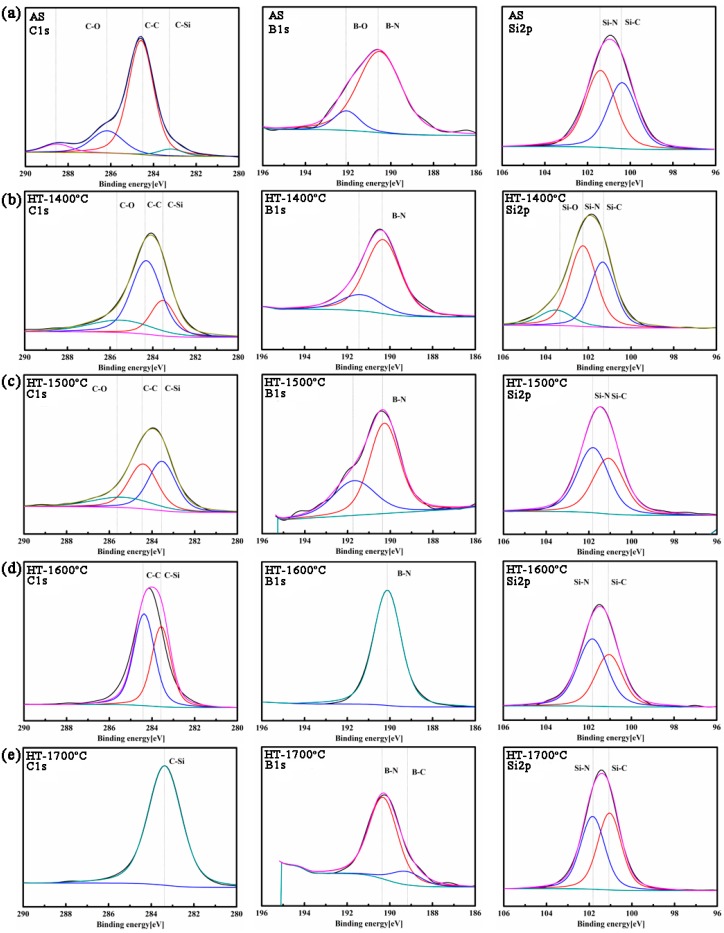
High-resolution XPS spectra of C1s, B1s, and Si2p core levels of SiBCN ceramics: (**a**) AS; (**b**) HT-14; (**c**) HT-15; (**d**) HT-16; and (**e**) HT-17.

XPS analysis was carried out to confirm the phase evolution and phase-reaction of SiBCN ceramics annealed at high temperatures. Figure 5 shows the narrow scanning spectra of C1s, B1s, and Si2p of SiBCN film before and after heat treatment. The correction of the XPS spectra was performed using C1s peak (E_B_ = 284.6 eV). For as-received SiBCN, the B1s spectrum was divided into two spectra, i.e., B–N at 191.1 eV and B–O at 193 eV [29]. The C1s peak was fitted into four peaks which were C-Si around 283.4 eV, free carbon at 284.6 eV, and C-O at 286.0 eV [30]. The relative content of C-C bonds was much higher than that of C-Si bonds, which was in reasonable agreement of the separation of amorphous carbon at 1400 °C. The Si2p peak could be decomposed into two sub peaks centered at 101.4 eV–102.0 eV and 100.4 eV, which should be assigned to Si–N [30] and Si–C [31] bonds, respectively. B–O and C–O bonds vanished simultaneously after annealing at 1600 °C, which confirmed that the slight weight loss of SiBCN-Si_3_N_4_ composite ceramics annealed before 1600 °C came from the absorption of the impurities in air. After further annealing at 1700 °C, the B–C bonds appeared though its relative content was much lower than B-N bonds, while the C–C bonds disappeared completely, only C–Si bonds remained. The bonds evolution agreed well with the results of XRD and Raman spectra, providing a certain evidence for phase evolution and the occurrence of phase-reaction. 

The relative complex permittivity (ε*_r_* = ε*′* − jε*″*) was measured in the frequency range of 8.2–12.4 GHz. According to the Debye theory, the real part of permittivity (ε′) is related to the polarization relaxation and the imaginary part of permittivity (ε″) represents the dielectric loss capability [15]. In addition, in order to fabricate the non-magnetic materials with excellent microwave absorption properties, the dielectric constant (real part of the permittivity) should be close to 1, and the dielectric loss (conductivity or imaginary part of the permittivity) should be high enough. Thereby, the dielectric loss (tanδ = ε″/ε′) can represent the EMW attenuation capacity of non-magnetic materials. This means that higher tanδ results in better microwave attenuation capability.

**Figure 6:**
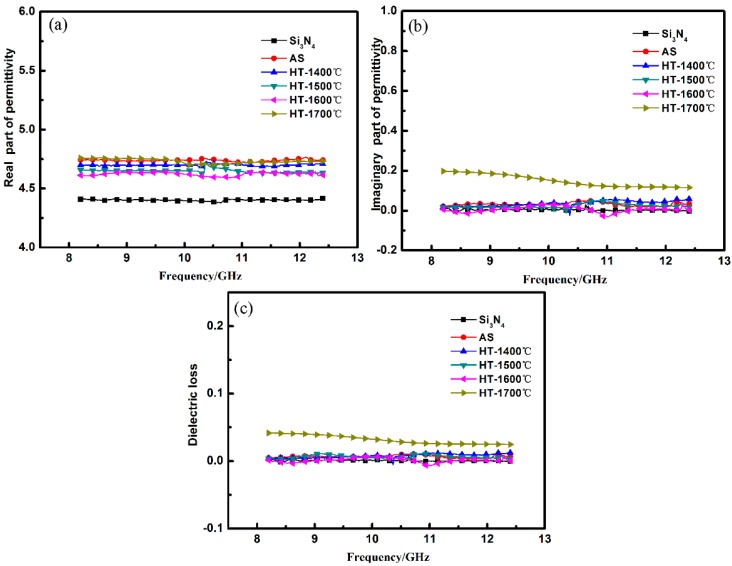
The permittivity of as-received and annealed SiBCN-Si_3_N_4_ ceramics at different temperatures: (**a**) the real part; (**b**) the imaginary part; and (**c**) tangent loss.

As shown in Figure 6, the complex permittivity of as-fabricated Si_3_N_4_ substrates was 4.4-j0 and stayed a constant after annealing at 1400–1700 °C due to the unchanged phases and microstructures. The Si_3_N_4_ substrates were exact insulators, so the complex permittivity of SiBCN-Si_3_N_4_ composite ceramics was determined mostly by the deposited SiBCN. The average ε′ and ε″ of AS were 4.74 and 0.02, respectively, and the dielectric loss was 0. That was in accordance with the results in our previous work [24] that the SiBCN fabricated at 800 °C was insulating due to the complete amorphous microstructure. After annealing at 1400–1700 °C, there was a slight variation in the average ε′. As for the average ε″, it kept a relatively stable value of around 0 after annealing at 1400–1600 °C while exhibiting an increase to 0.15 with the temperature rising to 1700 °C, and the dielectric loss accordingly increased to 0.03. 

According to above analysis about phase evolution and solid-phase reaction, the separated amorphous free carbon had no contribution to the complex permittivity of SiBCN ceramics after annealing at 1400–1600 °C. However, the products of solid-phase reaction after annealing at 1700 °C, SiC, had a strong dissipation capacity to EMW. SiC can become the dipole and the dipole will steer under the electric field and arrange regularly in the field direction. The dipolar polarization will take a long relaxation time and attenuate much energy. Moreover, the interface charge polarization between SiC and the amorphous phase also need long relaxation times, which can reduce the EMW energy effectively.

**Figure 7:**
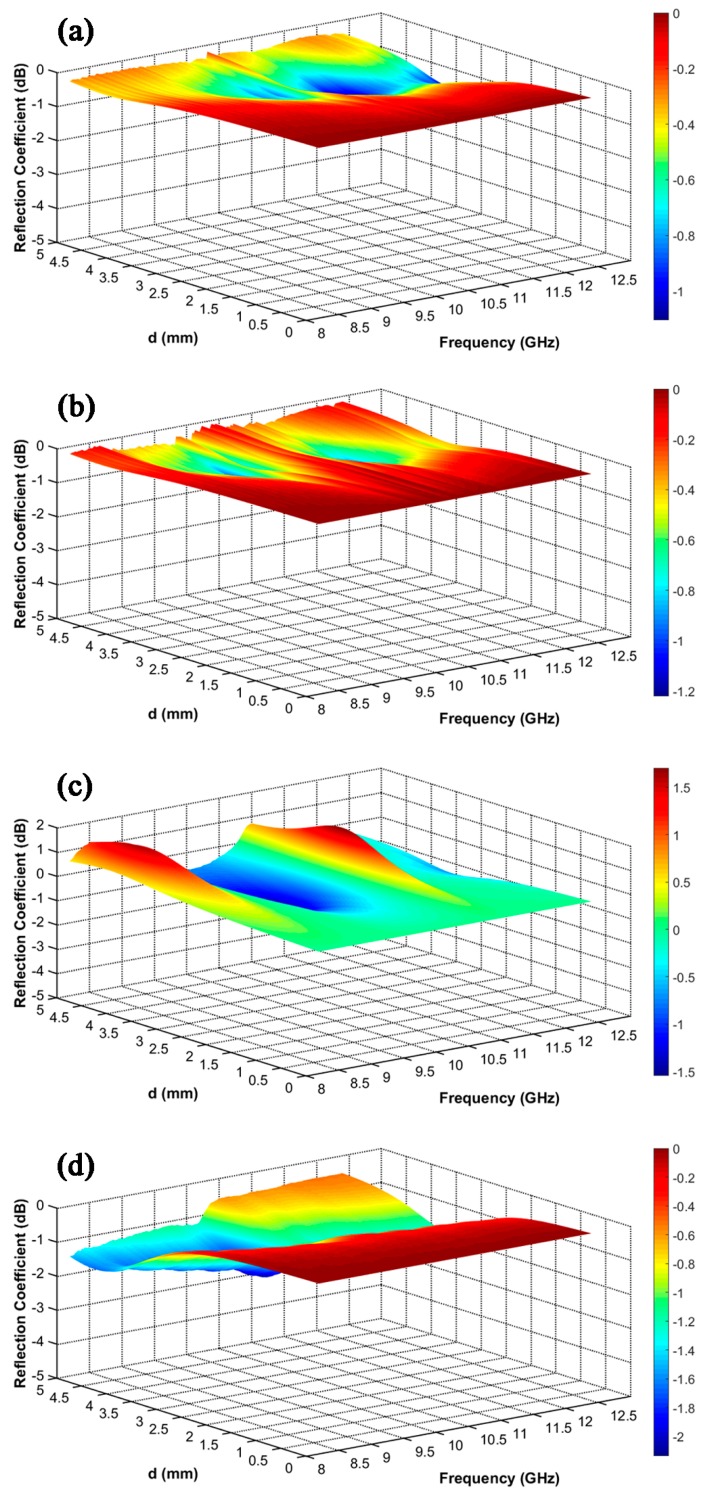
Reflection coefficient of SiBCN-Si_3_N_4_ ceramics heat-treated by different temperatures with different sample thicknesses in the frequency range of 8.2–12.4 GHz: (**a**) HT-14; (**b**) HT-15; (**c**) HT-16; and (**d**) HT-17.

As an important parameter for exhibiting the absorption properties of SiBCN ceramics, the reflection coefficient (*RC*) can be calculated according to the transmission line theory as follows, based on the metal back-panel model [32,33]:

(3) $$RC\left(\mathrm{dB}\right)=20\log\left|\frac{Z_{\mathrm{in}}-1}{Z_{\mathrm{in}}+1}\right|$$

where the normalized input impedance (*Z*_in_) is given by the formula:

(4) $$Z_{\mathrm{in}}=\sqrt{\frac{\mu_{r}}{\varepsilon_{r}}}\tan h\left[j\left(\frac{2\pi fd}{c}\right)\sqrt{\mu_{r}\varepsilon_{r}}\right]$$

where *ε_r_* = *ε′* − j*ε″*, *μ_r_* = *μ′* − j*μ″*, *f* is the EMW frequency (Hz), *d* is the thickness of the absorber (m), and *c* is the velocity of light in free space (m/s). Herein, *μ_r_* is taken as 1 because of the negligible magnetic properties of the SiBCN ceramics. *RC* of SiBCN-Si_3_N_4_ ceramics heat-treated by different temperatures with different sample thicknesses in the frequency range of 8.2–12.4 GHz are shown in Figure 7. 

SiBCN-Si_3_N_4_ ceramics showed higher *RC* after annealing from 1400 to 1700 °C, with a minimum *RC* of ca. −2 dB. Although the imaginary part of the permittivity of SiBCN-Si_3_N_4_ increased after annealing at 1700 °C, it was still at a low level. That is why *RC* of SiBCN-Si_3_N_4_ ceramics stayed so high. *RC* of SiBCN ceramics [16] fabricated by pyrolysis of liquid polyborosilazane gradually decreased with the annealing temperature increasing, and reached −15.78 dB at a thickness of 2.31 mm when annealed at 1650 °C. In our previous study of SiBCN ceramics deposited at 900 °C [24], SiBCN-Si_3_N_4_ ceramics with a dielectric loss of 0.1 showed proper dielectric properties. We want to study whether SiBCN ceramics deposited at lower temperature can also attain a better dielectric property after heat treatment. However, amounts of SiBCN introduced in SiBCN-Si_3_N_4_ at 800 °C was small and the free carbon was consumed after annealing at 1700 °C. Additionally, the amount of SiC formed was very small. All led to the lower EMW absorption property of the final SiBCN-Si_3_N_4_ composite ceramics.

Compared with the as-fabricated SiBCN ceramics, the ceramics annealed at 1700 °C can obtain different dielectric property, which can be attributed to the generation of SiC by phase-reaction. This work helped to further understand the microstructures of SiBCN ceramics. Since the element content and phase composition of SiBCN can be optimized by controlling the deposition parameters, including the ratios of precursor gases, the loss phase SiC can be separated by annealing at high temperature.

## 4. Conclusions

SiBCN-Si_3_N_4_ composite ceramics fabricated by introducing SiBCN into porous Si_3_N_4_ substrates were annealed at 1400–1700 °C. Amorphous free carbon, BN, and α-Si_3_N_4_ began to be separated at 1400 °C and 1600 °C, respectively. β-SiC began to be separated at 1500 °C. When the temperature increased to 1700 °C, more β-SiC formed by the reaction between free carbon and α-Si_3_N_4_. Consequently, the dielectric loss of composite ceramics increased to 0.03 from 0 due to the uniformly distributed SiC grains and the grain boundary.
